# HMGB1 as a Key Modulator in Nasal Inflammatory Disorders: A Narrative Review

**DOI:** 10.3390/jcm14155392

**Published:** 2025-07-31

**Authors:** Desiderio Passali, Luisa Maria Bellussi, Mariaconsiglia Santantonio, Giulio Cesare Passali

**Affiliations:** 1 ENT Clinic, University of Siena, 53100 Siena, SI, Italy; d.passali@virgilio.it (D.P.); l.bellussi@virgilio.it (L.M.B.); 2 Complex Operational Unit of Ear, Nose and Throat Sciences, Fondazione Policlinico Universitario A. Gemelli, Istituto di Ricovero e Cura a Carattere Scientifico (IRCCS), 00168 Rome, Italy; giuliocesare.passali@unicatt.it

**Keywords:** HMGB1, rhinitis, chronic rhinosinusitis, allergic rhinitis, glycyrrhizin

## Abstract

**Background**: High Mobility Group Box 1 is a mediator in inflammation, acting as a damage-associated molecular pattern molecule in various diseases. This review examines its role in nasal inflammatory disorders, such as chronic rhinosinusitis and allergic rhinitis. **Methods**: A comprehensive review of recent literature was conducted using a refined PubMed search strategy, focusing on studies published from 2015 onward and targeting HMGB1’s role in nasal inflammatory diseases. **Results**: HMGB1 emerges as a central factor in amplifying and modulating inflammatory responses through interactions with multiple receptors. It regulates cytokine production, epithelial–mesenchymal transition, and tissue remodeling, particularly in eosinophilic CRS. While discrepancies in the literature highlight its context-dependent activity, therapeutic strategies like glycyrrhetinic acid and PPAR-γ agonists demonstrate potential in modulating its effects. **Conclusions**: HMGB1 represents a promising diagnostic biomarker and therapeutic target in nasal inflammatory diseases. However, due to its intrinsic nature and multiple localizations, much remains to be understood. It is precisely by reflecting on its role as an “inflammatory crossroads” that we aim to underscore the need for targeted translational research to elucidate the molecular mechanisms and therapeutic applications of HMGB1.

## 1. Introduction

### 1.1. HMGB1 Overview

High Mobility Group Box 1 (also known as HMGB1, amphoterin, differentiation enhancing factor, sulphoglucuronyl carbohydrate binding protein-1, p30) [1] was first described in 1973 by Ernest Johns and colleagues as part of a group of non-histone proteins extracted from calf thymus chromatin, named “High Mobility Group” due to their rapid migration in polyacrylamide gel electrophoresis.

It is a highly conserved nuclear protein, widely expressed in mammalian cells, whose functions depend on its localization.

In the nucleus, HMGB1 acts as a DNA chaperone [2,3], stabilizing chromosomal structure [4] and regulating gene transcription. However, under stress conditions, HMGB1 can translocate to the cytoplasm or be released into the extracellular space, either actively or passively.

### 1.2. HMGB1 in the Extracellular Space

In the extracellular environment, it functions as a damage-associated molecular pattern (DAMP) molecule, activating inflammatory and immune responses through receptors like TLR4 and RAGE (Receptor for Advanced Glycation End Products). The innate immune system relies on pattern-recognition receptors (PRRs) like Toll-like receptors (TLRs) and RAGE to detect pathogens or cellular damage. These receptors share ligands such as HMGB1, S100A8/A9, and lipopolysaccharides (LPS), and their interactions play a key role in immune responses, though the mechanisms behind their signaling and cooperation remain unclear [5]. While other PRRs such as NOD-like receptors (NLRs) and C-type lectin receptors (CLRs) also play roles in innate immunity, their interaction with HMGB1 has been less extensively studied.

Furthermore, HMGB1 has been proven to drive angiogenesis by inducing proangiogenic cytokine release and activating endothelial cells, macrophages, EPCs, and mesoangioblasts, contributing to vessel formation [6].

This dual role makes HMGB1 a mediator of inflammation and a plausible biomarker in various diseases [7,8,9,10,11,12,13].

### 1.3. HMGB1 Modifications and Functions

During cellular stress, HMGB1 undergoes a tightly regulated shift in localization and activity [14,15], orchestrated by post-translational modifications such as acetylation, phosphorylation, methylation, and oxidation [16]. These modifications affect its nuclear localization signals (NLS), allowing HMGB1 to exit the nucleus and accumulate in the cytoplasm or extracellular milieu. HMGB1 release occurs passively during cell death (e.g., necrosis or pyroptosis) or actively in response to stress signals like LPS [17,18]. Active secretion follows non-classical pathways, such as lysosomal exocytosis, bypassing the conventional endoplasmic reticulum–Golgi route.

Reactive oxygen and nitrogen species (ROS and RNS) further modulate its activity by influencing the redox state: fully reduced HMGB1 interacts with CXCL12 to promote chemotaxis via CXCR4 and supports cell recruitment and tissue regeneration [19,20]; disulfide HMGB1 stimulates TLR4-dependent cytokine production and promotes inflammation; overoxidized HMGB1 acts as an immune suppressor.

In this context, RAGE is a crucial player in HMGB1-induced inflammation [21,22,23,24,25]. It binds HMGB1 and mediates its internalization via endocytosis, facilitating the transport of HMGB1 and its complexes (e.g., with DNA, RNA, or lipopolysaccharides) into the cytoplasm, where these molecules interact with intracellular receptors like TLR9, AIM2, or caspase-11. This process sustains inflammatory responses in conditions such as sepsis, chronic inflammation, and autoimmune diseases by activating transcription factors, such as NF-kB, which drives the production of pro-inflammatory cytokines.

Several studies have also highlighted the role of HMGB1 in endothelial cell activation, a key event in systemic inflammation and sepsis [26,27,28,29]. HMGB1 induces the expression of adhesion molecules such as ICAM-1 and VCAM-1 on endothelial cells, promoting leukocyte adhesion and migration into inflamed tissues. It also stimulates the secretion of pro-inflammatory cytokines (e.g., TNF-α) and chemokines (e.g., IL-8 and MCP-1) [30], amplifying the inflammatory response.

HMGB1 also influences the fibrinolytic system by enhancing the release of tissue plasminogen activator (tPA) and plasminogen activator inhibitor-1 (PAI-1), which modulate coagulation and fibrinolysis. These actions, mediated in part through RAGE and MAP kinase pathways (e.g., ERK, JNK, p38), underscore HMGB1’s central role in endothelial dysfunction during inflammation. Furthermore, HMGB1 interacts with TLR9 [31,32] by forming complexes with DNA, promoting TLR9 translocation to early endosomes where it triggers cytokine release through MyD88- and NF-κB-dependent pathways. This interaction amplifies the production of IL-1, IL-6, and TNF-α and enhances immune activation, highlighting HMGB1’s role in early inflammatory responses.

In addition, HMGB1 has been implicated in a mechanism of pyroptosis, a pro-inflammatory form of programmed cell death. In hyperhomocysteinemia (HHcy), HMGB1 promotes endothelial pyroptosis by increasing lysosomal permeability and releasing cathepsin V, a lysosomal cysteine protease. Cathepsin V activates the NLRP3 inflammasome and caspase-1, leading to the cleavage of gasdermin D (GSDMD) and subsequent IL-1β release. Inhibition of cathepsin V or silencing HMGB1 effectively rescues endothelial cells from Hcy-induced pyroptosis, highlighting their interconnected roles.

Overall, due to its central role in driving inflammation, HMGB1 has emerged as a promising therapeutic focus [33]. Its interactions with TLR2 [34], TLR4, TLR 9 and RAGE not only modulate inflammatory cascades but also facilitate the formation of pro-inflammatory complexes with other molecules such as DNA, RNA, and LPS, amplifying its pathological effects in conditions like chronic inflammation, sepsis, and tissue damage.

**Figure 1:**
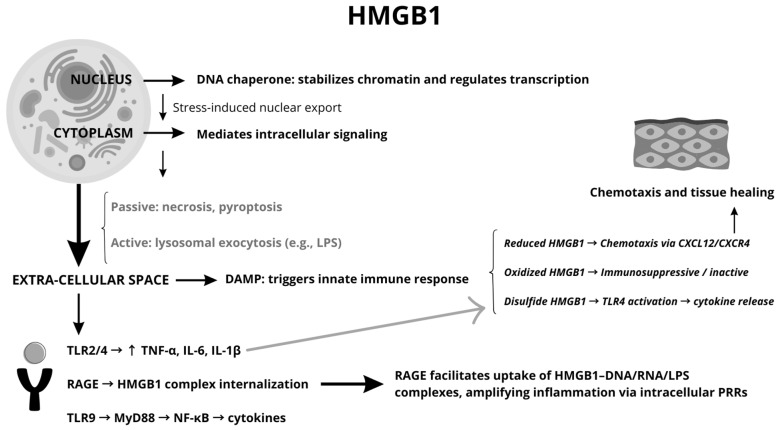
Schematic representation of HMGB1 signaling pathways.

A summary diagram of the functions, localizations, and molecules involved in the HMGB1 pathway is provided in Table 1 and Figure 1.

### 1.4. CRS Overview

CRS is classified into two main phenotypes, CRS with nasal polyps (CRSwNP) and CRS without nasal polyps (CRSsNP), but emerging evidence identifies distinct endotypes based on immune response patterns [35,36]. These include type 1 (Th1, Tc1, ILC1), type 2 (Th2, Tc2, ILC2), and type 3 (Th17, Tc17, ILC3) immune responses. Type-2 immune responses, characterized by elevated IL-4, IL-5, IL-13, and local IgE production, are strongly associated with eosinophilic inflammation, nasal polyp formation, asthma comorbidity, and severe disease [37,38,39]. Neutrophils are highly abundant in both eosinophilic and non-eosinophilic CRS but play a more prominent role in non-eosinophilic CRS and steroid-resistant disease phenotypes [40]. Neutrophil-driven inflammation is linked to poor corticosteroid responses and disease persistence, particularly in Asian populations, where neutrophilic CRS is more prevalent. Key mechanisms include the recruitment of neutrophils via chemokines (e.g., CXCL1, CXCL8) and IL-17A, which sustain neutrophilic infiltration and amplify local inflammation. Neutrophils also contribute to epithelial barrier dysfunction, tissue remodeling, and the release of extracellular traps (NETs), further exacerbating CRS severity. It is worth noting that approximately 80% of nasal polyps in patients with CRSwNP in the western countries exhibit eosinophilic inflammation, typically associated with type-2 immune responses [35,36,37,38].

As discussed in the Review of Literature section, HMGB1 appears to play an important role in CRS by amplifying both eosinophil- and neutrophil-driven inflammation. Its activity may influence epithelial barrier dysfunction, immune cell recruitment, and the persistence of inflammation, contributing to the distinct immune endotypes observed in CRS [18].

### 1.5. Allergic Rhinitis Overview

Allergic rhinitis (AR) is a prevalent chronic inflammatory condition of the nasal mucosa characterized by symptoms such as sneezing, nasal congestion, runny nose, and itching, often accompanied by watery eyes and an itchy throat. It is a type-I hypersensitivity reaction mediated by IgE, triggered by exposure to allergens [41,42].

The pathogenesis of AR involves multiple factors, including nasal epithelial barrier dysfunction, which facilitates allergen penetration and immune activation. This triggers a Th2-dominant immune response, characterized by the overproduction of cytokines like IL-4, IL-5, and IL-13, leading to inflammation and IgE production. Subsequent allergen exposure activates mast cells via IgE, causing the release of histamine, leukotrienes, and prostaglandins, which drive AR symptoms [43]. Current treatments focus on symptom control through antihistamines, corticosteroids, and immunotherapy, though long-term efficacy remains limited, underscoring the need for novel therapeutic approaches.

This review aims to explore the role of HMGB1 in nasal inflammatory diseases, ranging from AR and non-specific rhinitis to CRSwNP. These conditions, while clinically distinct, share a common underlying inflammatory ecosystem that often remains obscured beneath their diverse presentations. HMGB1, as a mediator of inflammation, emerges as a potential unifying factor in these diseases [44]. Its ability to act as a DAMP molecule and amplify inflammatory cascades suggests a pivotal role in sustaining the chronic inflammation characteristic of these conditions.

## 2. Materials and Methods

### 2.1. Research and Screening of Literature

The literature search was conducted on PubMed using the following search strategy: HMGB1 AND (“nasal polyps” OR “nasal polyposis” OR “rhinitis” OR “allergic rhinitis” OR “rhinosinusitis” OR “chronic rhinosinusitis” OR “crswnp” OR “chronic rhinosinusitis with nasal polyps” OR “nasal epithelium” OR “respiratory epithelium”).

The search strategy for this review was refined by adding specific terms to better capture the range of nasal inflammatory diseases associated with HMGB1.

The first filter applied in the research, alongside the use of specific keywords, was the temporal span. To ensure the inclusion of the most up-to-date and relevant findings, we restricted our literature search to articles published from 2015 onward. This ten-year time frame was chosen to reflect recent advances in the understanding of HMGB1 biology, particularly its redox-dependent mechanisms, receptor interactions, and potential as a therapeutic target in upper airway inflammatory diseases.

After the initial search yielded 53 results, duplicate and irrelevant articles were excluded following title and abstract screening, which were independently screened by two reviewers to determine relevance. Discrepancies between reviewers were resolved through discussion or consultation with a third reviewer. The remaining studies were assessed through full-text analysis, leading to the inclusion of 29 of them in the review.

For a more homogeneous description of the results, the articles were divided into four categories, based on the main topic relating to the evaluation of HMGB1: CRS, AR, both CRS and AR and non-specific rhinitis or nasal inflammatory conditions not falling into the previous categories.

**Figure 2:**
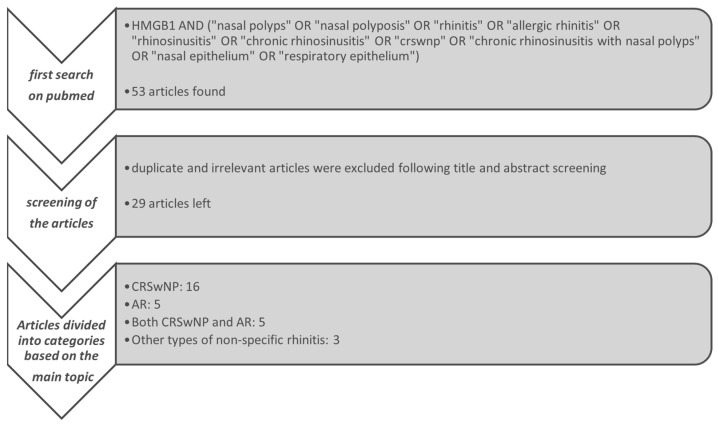
Flowchart of the study selection process. The figure summarizes the literature search strategy, screening steps and the categories selected.

The entire process of searching, screening and categorizing is shown graphically in Figure 2.

### 2.2. Inclusion and Exclusion Criteria

The inclusion criteria for this review were as follows: studies published from 2015 onward, indexed in databases such as PubMed or other relevant sources, and specifically investigating the role of HMGB1 in nasal inflammatory diseases, including conditions such as chronic rhinosinusitis, nasal polyposis, and rhinitis. The exclusion criteria included duplicate studies, articles not directly relevant to the topic (excluded after screening titles and abstracts) and research focused on animal models.

## 3. Results

### 3.1. HMGB1 and CRS

Several studies included in this section have explored the role of HMGB1 in the pathogenesis of CRS, employing diverse methodologies, including proteomic analyses, immunohistochemistry, ELISA, Western blot, and cell culture experiments. These studies examined CRS with and without nasal polyps, with sample sizes ranging from 8 to 63 patients/samples per study, and in vitro models utilizing epithelial cells and fibroblasts from nasal tissues.

A consistent finding across studies is the increased expression and extracellular release of HMGB1 in CRS tissues and nasal secretions, particularly in eosinophilic CRSwNP [45,46,47], and its contribute to inflammation, epithelial–mesenchymal transition (EMT), and tissue remodeling [48,49,50] through its interaction with RAGE and TLR4/9 signaling pathways, promoting cytokine release (IL-6, IL-8, TNF-α) and immune cell recruitment.

Additionally, studies have suggested that HMGB1-driven inflammation correlates with disease severity and could serve as a biomarker to distinguish eosinophilic from non-eosinophilic CRS phenotypes.

RAGE overexpression itself has been evaluated as a marker of disease severity in CRSwNP [51] and may contribute to mucosal hyperproliferation and repeated polyp formation. A study from 2015 [52], through immunohistochemistry and ELISA analysis, evaluated HMGB1 and RAGE expression in 37 CRS patients and 26 controls, assessing disease severity through SNOT-20 questionnaires, nasal endoscopy, and CT scans. While HMGB1 immuno-expression showed no significant differences between groups, RAGE expression was markedly higher in CRS patients and correlated positively with lymphocyte infiltration, disease severity, and allergy history. This suggests that HMGB1 alone may not contribute as a key factor in CRS pathogenesis and RAGE, instead, seems to play a more significant role in this context.

Multiple studies have underscored the connection between HMGB1 levels and inflammation [53,54,55]. For instance, it was demonstrated a positive correlation between HMGB1 levels in nasal lavage fluid and inflammation severity, as measured by the Lund-Mackay score [53]. Similarly, elevated HMGB1 and TLR4 expression were found in CRSwNP patients compared to controls, with a strong positive correlation between these markers [54]. These findings suggest that HMGB1, as a ligand for TLR4, amplifies immune responses and cytokine production, contributing to chronic inflammation and epithelial dysfunction.

Proteomics in CRS research has further advanced biomarker discovery. Pesold and colleagues in 2023 [45] identified seven proteins, including HMGB1, differentially expressed in nasal mucus from CRSsNP patients compared to controls. These proteins, associated with inflammation, apoptosis, and cell–matrix interactions, were validated across multiple cohorts, providing insight into CRSsNP endotyping.

Another study was conducted in vitro on human nasal epithelial cells (HNECs), explored the role of miR-1287-5p in CRS [56]. In this study the samples included normal nasal mucosa samples and six samples from patients with CRS. MiR-1287-5p, significantly downregulated in CRS patients, was shown to inhibit SNAI1 and HMGB1, thereby reducing pro-inflammatory cytokines (IL-6, IL-8, TNF-α) and EMT [57,58,59]. Upregulation of miR-1287-5p reduced HMGB1 expression at both the mRNA and protein levels. Bioinformatics analysis and dual-luciferase reporter assays confirmed that miR-1287-5p directly targets and inhibits SNAI1. Knocking down SNAI1 further suppressed HMGB1 expression, limiting both inflammation and EMT. Similarly, the HMGB1 inhibitor Glycyrrhizin reduced SNAI1 expression, mitigating EMT.

Necroptosis, a form of programmed cell death, has also been implicated in CRSwNP pathogenesis [48]. Markers of necroptosis, such as phosphorylated RIPK3 and MLKL, were elevated in eosinophilic and non-eosinophilic CRSwNP, particularly in macrophages. Necroptosis promotes the release of DAMPs, including HMGB1 and IL-1α, which amplify neutrophilic inflammation through cytokines such as IL-8 and CXCL1. The mTOR pathway mediates this process, triggered by TNF-α and IFN-γ. Furthermore, the type of inflammation may influence HMGB1 expression. A study from 2021 [60] reported significantly higher HMGB1 levels in ECRSwNP compared to non-eosinophilic forms, with HMGB1 levels effectively distinguishing between these subtypes. This highlights HMGB1 as a potential biomarker and therapeutic target for ECRSwNP.

A 2023 study [61] explored the regulatory role of HMGB1, revealing its interaction with the RAGE-MEK pathway as a driver of elevated 15-hydroxyprostaglandin dehydrogenase (HPGD) expression. Through a retrospective analysis of 40 patients divided into three groups (control, NECRSwNP, and ECRSwNP), the study found that this upregulation reduced prostaglandin E2 (PGE2) levels, thereby impairing inflammation resolution in CRSwNP. In vitro experiments confirmed that recombinant HMGB1 stimulated HPGD expression in primary human nasal epithelial cells in a time-dependent manner. Activation of the RAGE-MEK signaling pathway contributed to this effect, while RAGE inhibition partially blocked it, stressing its potential as a therapeutic target.

Moreover, TLR9-mediated pathways may play a role in CRSwNP pathogenesis, as evidenced by a study [62] showing that TLR9 activation enhances BAFF (B-cell Activating Factor) production and stimulates type-I interferons. The study enrolled 45 patients and included an in vitro investigation using cultured dispersed nasal polyp cells (DNPCs) to assess the effects of TLR9 activation on HMGB1 and immune response. Immunofluorescence analysis revealed a higher co-expression of HMGB1 and TLR9 in CRSwNP patients. Notably, the TLR9-HMGB1-IFN-BAFF pathway emerged, from this study, as a therapeutic target, with chloroquine (a TLR9 inhibitor) effectively reducing BAFF production, therefore mitigating inflammation.

Potential therapeutic strategies targeting HMGB1 include glycyrrhetinic acid (GA) and peroxisome proliferator-activated receptor-γ (PPAR-γ) agonists like rosiglitazone, which reduce HMGB1 activity, EMT, and inflammation [58,63,64].

Specifically, Sirt6 expression, found in normal nasal mucosa, is significantly reduced in CRSwNP tissues, and its depletion leads to HMGB1 translocation from the nucleus to the cytoplasm, contributing to inflammation. GA, a compound derived from licorice, has demonstrated anti-inflammatory properties by enhancing Sirt6 expression, inhibiting HMGB1 translocation, and reducing its extracellular accumulation [65]. A study from 2019 explored the role of HMGB1 in driving EMT in eosinophilic chronic rhinosinusitis with nasal polyps (ECRSwNP) whilst also highlighting the therapeutic potential of peroxisome proliferator-activated receptor-γ (PPAR-γ) agonists [58]. Eighteen ECRSwNP tissue samples and twelve normal nasal mucosa samples were obtained and diagnoses were confirmed histologically; HMGB1 was found to be highly expressed and localized in the cytoplasm of ECRSwNP tissues, promoting EMT by downregulating epithelial markers (E-cadherin, ZO-1) and upregulating mesenchymal markers (vimentin, N-cadherin). Rosiglitazone (ROG), a PPAR-γ agonist, effectively inhibited HMGB1-induced EMT by restoring epithelial marker expression and suppressing mesenchymal markers. Additionally, ROG reduced LPS-induced HMGB1 release, demonstrating its ability to modulate both extracellular and endogenous HMGB1 activity.

It is important to point out that both glycyrrhizin and glycyrrhetinic acid are bioactive compounds derived from licorice (Glycyrrhiza glabra), yet they differ in structure and function. Glycyrrhizin is a natural saponin that directly binds HMGB1, while glycyrrhetinic acid (GA), its hydrolyzed metabolite, exerts broader anti-inflammatory effects, including modulation of HMGB1 signaling pathways.

The studies summarized in this section highlight HMGB1 as a mediator in CRS, with potential implications for diagnostics and therapeutics. These findings are consolidated in Table 2.

### 3.2. HMGB1 and AR

HMGB1 has emerged as a pro-inflammatory mediator in the pathogenesis of AR [43]. It contributes to AR by disrupting the nasal epithelial barrier, promoting allergen penetration, and triggering immune responses.

It binds to receptors like RAGE and TLR4, activating pathways (e.g., NF-κB, MAPK) that drive inflammation and cytokine release (e.g., IL-4, IL-5, IL-13, IL-17A). HMGB1 also recruits immune cells like eosinophils and amplifies Th2/Th17 polarization, exacerbating AR symptoms.

Immunohistochemical analysis has revealed significantly elevated levels of HMGB1 in the nasal mucosa of AR patients compared to controls, with its expression being closely linked to IL-33, as both show a time- and dose-dependent increase when stimulated with allergens like Der p 1 [67].

A study from 2020 explored the role of HMGB1 and TLR4 in AR by analyzing 125 AR patients and 87 healthy controls. AR patients exhibited elevated levels of IL-4, IL-5, IL-13, and IL-17A in nasal lavage fluid, along with reduced IL-10 levels. Increased mRNA expression of HMGB1, TLR2, and TLR4 was observed in nasal brushing samples from AR patients, while TLR3 and RAGE showed no significant differences. HMGB1 and TLR4 levels positively correlated with pro-inflammatory cytokines (IL-4, IL-5, IL-13, IL-17A) and negatively with IL-10. These findings suggest that the HMGB1/TLR4 signaling pathway may contribute to AR pathogenesis and could represent a potential target for immunotherapy [68]. Serum HMGB1 levels are also seem to be associated with elevated inflammatory markers (e.g., IL-6, CRP) and correlate with clinical symptom severity [69]. Moreover, HMGB1 appears to be a target of miR-141-3p, which suppresses HMGB1 expression and reduces mucin production and cell apoptosis in LPS-treated nasal epithelial cells, pointing to a regulatory axis that may be therapeutically exploitable [70]. Lastly, HMGB1 contributes to epithelial barrier dysfunction and immune dysregulation in AR, with inhibitors like GA and ethyl pyruvate showing promise in reducing inflammation in AR models [35].

The results of the studies included in this section are summarized in Table 3.

### 3.3. HMGB1 and Both CRS and AR

As already stated, HMGB1’s activity can be modulated by GA, which has demonstrated anti-inflammatory effects by reducing HMGB1 levels and improving clinical symptoms, positioning HMGB1 modulation as a therapeutic strategy [49].

Similarly, HMGB1 amplifies inflammatory responses through TLR4 signaling, promoting the secretion of IL-6 and IL-8, further implicating its role in upper airway inflammation [59]. Eosinophils are identified as a primary source of extracellular HMGB1 in AR and related disorders, with glycyrrhizin (GLT) effectively reducing HMGB1 levels and selectively inducing eosinophil apoptosis, thereby mitigating inflammation [63]. Systematic reviews confirm HMGB1 as a pivotal inflammatory mediator, with GA showing efficacy comparable to corticosteroids but with a superior safety profile, underscoring its potential in clinical applications [50]. However, at physiological levels, HMGB1 does not seem to independently affect eosinophil survival or chemotaxis, suggesting that its pathological role is context-dependent and influenced by interactions with other inflammatory mediators [47].

The pathways and cytokines involved in the inflammation present in CRS and AR, as well as their connection to HMGB1, are summarized in Table 4.

### 3.4. HMGB1 and Non-Specific Nasal Inflammatory Diseases

HMGB1 plays roles in general nasal epithelial dysfunction [71], specifically—as some studies highlight—under hypoxia [66,72], mediates necroptosis-driven inflammation, and regulates immune responses in non-specific inflammatory conditions [48,66]. A study from 2016 furtherly stressed the hypoxia-driven HMGB1 activation: a translational experimental study combining in vitro models (using human nasal epithelial cells, NHNE) and ex vivo/in vivo analyses (human nasal mucosa and lavage samples), which investigated how hypoxia induces HMGB1 secretion through a ROS-dependent mechanism driven by DUOX2.

Under hypoxic conditions, HMGB1 translocates from the nucleus to the extracellular space, where it promotes the secretion of IL-8, a pro-inflammatory cytokine. The findings were validated in patient samples, showing increased HMGB1 and IL-8 levels in hypoxic nasal mucosa and lavage fluids [73].

Now, reinforcing the notion of HMGB1’s diverse roles, the idea that its function depends on context or interactions with other mediators fits precisely within the framework of balance [47]. This last paper in particular challenges the role of HMGB1 as a direct mediator in nasal inflammation. It found that physiological and even pathophysiological concentrations of HMGB1 had no effect on eosinophil survival or chemotaxis, suggesting that HMGB1 alone does not directly contribute to eosinophil-related inflammation. Most studies highlight the role of HMGB1 in complex inflammatory pathways or its interaction with other molecules, such as cytokines or DAMPs. Dyer et al.‘s findings emphasize that HMGB1 likely acts as part of a larger molecular network, rather than as a standalone driver of inflammation.

A summary of the common findings across different studies is presented in Table 5.

## 4. Discussion

We conducted a comprehensive PubMed search for “HMGB1,” retrieving 11,515 results, including 95 publications from 2025. After removing duplicates and studies without abstracts, we analyzed a final dataset of 8315 publications. Among these: 5147 studies involved animal models; 1612 were in vitro studies with no direct human application; 1266 were reviews or systematic reviews; 140 studies included cohorts of over 100 patients; 386 focused on small groups (fewer than 50 patients). The publication span (1964–2025) reflects the growing interest in HMGB1 research. However, the disproportionate focus on animal models—especially 50 of the 95 studies published in 2025—highlights a gap in large-scale human research, despite advancements in non-invasive sample collection methods.

### 4.1. Discrepant Results

Several studies have provided conflicting data regarding the role of HMGB1 in nasal inflammatory disorders. While HMGB1 levels in nasal lavage fluid were shown to correlate with inflammation severity in one study [53], another investigation [52] found no significant differences in HMGB1 expression between CRSsNP patients and healthy subjects, although RAGE expression was significantly elevated.

Very interestingly, other trials (non-related to the rhinology field) highlighted that HMGB1 has limited pro-inflammatory activity in its pure form, acting primarily as a chemoattractant and mitogen. However, when it forms complexes with molecules such as ssDNA, LPS, IL-1β, and nucleosomes, it becomes highly inflammatory by activating receptors like TLR9, TLR4, IL-1R, and TLR2 [74,75]. In AR patients, data on HMGB1 levels are less consistent. While some studies found increased expression during allergen exposure, others showed no significant change compared to healthy subjects.

Released by damaged or activated cells, HMGB1 serves as a danger signal originating from the body itself, playing a crucial role in both inflammation and tissue repair. This dual activity allows HMGB1 to balance the need to sacrifice or reconstruct tissues depending on the presence of pathogens or damage.

The overall findings on HMGB1 expression in upper airway inflammation are not entirely consistent across studies. While elevated levels are generally observed in CRSwNP, results in CRSsNP and AR are more variable, possibly reflecting differences in patient phenotyping, timing of sample collection, and methodological heterogeneity. Additionally, discrepancies in the reported cellular localization of HMGB1 highlight the need for standardized protocols to assess its compartmentalization and functional status.

### 4.2. Therapeutic Possibilities

#### 4.2.1. Preclinical Evidence for HMGB1 Inhibition

Several preclinical studies have identified HMGB1 as a promising therapeutic target in upper airway inflammation. GA, a natural compound with anti-inflammatory properties, has consistently demonstrated HMGB1-inhibitory effects, leading to reduced inflammation and improved clinical outcomes in both AR and CRS models [46,49]. Additional strategies have focused on modulation of Sirt6, which prevents HMGB1 translocation and mitigates its extracellular pro-inflammatory activity [65]. PPAR-γ agonists have also been shown to inhibit HMGB1-driven EMT in eosinophilic CRSwNP [58], further expanding the spectrum of potential pharmacological interventions.

#### 4.2.2. Challenges in Clinical Translation

Despite these encouraging findings, the translation of HMGB1-targeting therapies into clinical practice remains a challenge. One key limitation lies in the heterogeneous nature of both CRS and AR, which encompass multiple inflammatory endotypes. HMGB1 expression appears to vary significantly depending on disease subtype, particularly in eosinophilic versus non-eosinophilic forms. This variability underscores the need for stratified trial designs and biomarker-based patient selection to enhance therapeutic precision and reduce variability in treatment response.

#### 4.2.3. Key Considerations for Clinical Trial Design

Clinical trials aimed at evaluating HMGB1-targeting agents should incorporate several essential components:

1. The identification of reliable biomarkers, such as (potentially) HMGB1 levels in nasal lavage or tissue samples;
2. Standardized clinical outcome measures, including changes in SNOT-22 scores, polyp size, or levels of inflammatory cytokines; and
3. Clear inclusion criteria based on clinical phenotype and inflammatory profile.

Initial phase I/II trials may focus on safety, tolerability, and proof-of-mechanism endpoints, such as shifts in HMGB1 localization or downstream cytokine activity

#### 4.2.4. Future Perspectives

Future randomized controlled trials should aim to compare HMGB1-targeting therapies with current anti-inflammatory treatments, such as corticosteroids, potentially also exploring combination regimens. Given the pleiotropic mechanisms of GA, dose-finding studies will be critical to balance efficacy and safety, particularly in light of possible systemic effects. Long-term follow-up will be essential to evaluate sustained efficacy, recurrence rates, and the potential of HMGB1 inhibition to modify disease progression.

### 4.3. Challenges Due to Limited Sample Sizes

An important limitation in the reviewed studies is the small sample size, which reduces statistical power and generalizability. Many studies involved fewer than 50 participants, making it difficult to capture the heterogeneity of CRS and AR phenotypes. Additionally, subgroup analyses (e.g., eosinophilic vs. non-eosinophilic CRSwNP) often rely on small cohorts, complicating efforts to identify reliable biomarkers or therapeutic targets.

### 4.4. Spatiotemporal Dynamics and Mechanistic Implications of HMGB1 Action

From Section 3.1 HMGB1 and CRS, some intriguing insights can be extracted regarding the “where of this multifunctional protein. Specifically, it is interesting to note the number of studies that have confirmed the crucial role of the RAGE receptor, in some cases even surpassing the importance of HMGB1 itself.

And what about the “how”? How can we achieve meaningful results and effectively block this inflammatory intermediary? GA has shown results as a potential ally, yet much remains to be understood about its mechanisms of action. Does it work by reducing SNAI1 expression and mitigating EMT? By lowering HMGB1 levels and selectively inducing eosinophil apoptosis, thereby reducing inflammation? Or perhaps by enhancing Sirt6 expression, inhibiting HMGB1 translocation, and limiting its extracellular accumulation? All these pathways, studied separately, appear to have numerous points of convergence, many of which are directly driven by HMGB1 activation.

It is certainly interesting that the discrepancies and contradictions among different studies highlight how intrinsically HMGB1 activity is tied to multiple inflammatory cascades. This places HMGB1, both in terms of its position and function, intricately embedded within a complex network of other agents and receptors that shape its behavior.

## 5. Conclusions

We propose the following questions to stimulate future research directions, as we believe they provide a foundation for thought-provoking reflections.

Most clinical studies on HMGB1 in CRS and AR involve fewer than 50 patients, limiting their generalizability. How can standardized protocols and collaborative research networks ensure more robust, reproducible findings? Studies indicate that oxidized, disulfide, and reduced HMGB1 have distinct biological functions. Should future research focus on selectively modulating its redox state rather than blocking HMGB1 entirely? While HMGB1 has been consistently implicated in CRS and AR, some studies suggest it may function more as an amplifier rather than the primary initiator of inflammation. Could targeting its upstream regulators yield more effective therapeutic outcomes?

## Figures and Tables

**Table 1:** Summary of HMGB1 structural and functional properties relevant to upper airway inflammation. The table includes key features, molecular characteristics, and biological observations from current literature.

Feature	Description	Observations
**Discovery**	Identified in 1973 as a non-histone protein extracted from calf thymus chromatin; named ‘High Mobility Group’ for its high mobility in gel electrophoresis.	Significant for its rapid migration in electrophoresis and role in chromatin biology.
**Localization**	**Nucleus**: Functions as a DNA chaperone, maintains chromosomal structure, and regulates transcription. **Cytoplasm**: Interacts with cellular proteins. **Extracellular**: Acts as a damage-associated molecular pattern (DAMP)	Functions depend on its subcellular location; extracellular HMGB1 serves as an inflammatory modulator or mediator based on its interactions with the environment
**Release Mechanisms**	**Passive**: Released during cell death (e.g., necrosis, pyroptosis) as membrane integrity is lost. **Active**: Secreted via non-conventional pathways (e.g., lysosomal exocytosis) in response to stress signals like LPS.	Non-classical secretion pathways bypass the ER–Golgi system, ensuring release under stress conditions.
**Post-Translational Modifications**	Acetylation, phosphorylation, methylation, and oxidation regulate its translocation from the nucleus to the cytoplasm and extracellular space.	Post-translational modifications determine cellular localization and inflammatory activity of HMGB1.
**Redox States**	**Reduced**: Promotes chemotaxis by forming complexes with CXCL12. **Disulfide**: Activates TLR4-mediated inflammation. **Overoxidized**: Loses pro-inflammatory activity, acting as an immune suppressor.	The redox state is pivotal in defining HMGB1’s role in chemotaxis, cytokine activation, or immune suppression.
**Cytokines whose release is induced by HMGB1** [6,10]	Tumor Necrosis Factor (TNF) Interleukin-1 (IL-1) Interleukin-6 (IL-6) Interleukin-8 (IL-8) Macrophage Inflammatory Protein-1 (MIP-1) Monocyte Chemoattractant Protein-1 (MCP-1)	**TNF**: Drives inflammation, immune cell recruitment, and apoptosis. **IL-1**: Triggers fever, acute inflammation, and leukocyte recruitment. **IL-6**: Promotes acute-phase protein production and adaptive immunity. **IL-8**: Attracts neutrophils to inflammation sites. **MIP-1**: Recruits macrophages and other immune cells. **MCP-1**: Recruits monocytes, macrophages, and T-cells to sites of inflammation.
**Receptors**	**TLR 2 and 4**: Drives production of pro-inflammatory cytokines. **RAGE**: Facilitates endocytosis of HMGB1 and its complexes, amplifying inflammation by delivering cargo to intracellular receptors. **TLR9**: Triggers cytokine release through MyD88- and NF-κB-dependent pathways.	RAGE’s role in lysosomal disruption links extracellular signals to intracellular inflammatory pathways.
**Therapeutic Target**	Strategies include neutralizing antibodies, TLR4/RAGE inhibitors, and drugs targeting HMGB1 release or interactions. Delayed HMGB1 release offers a therapeutic window in inflammatory diseases.	Promising results in preclinical models for sepsis, chronic inflammation, and autoimmune conditions.

**Table 2:** Summary of studies investigating the role of HMGB1 in chronic rhinosinusitis (CRS), including study design, population, key findings, and clinical or molecular observations.

Study	Receptor Activity	Type of Study	N of Subjects	Correlation with Severity	Activated Cytokines	Key Findings
**Chen S et al., 2023 [61]**	RAGE-MEK pathway; upregulation of HPGD	Experimental; in vitro and ex vivo analysis	Control group: 9; NE CRSwNP: 18; ECRSwNP: 13;	Elevated HPGD correlates with CRSwNP severity	PGE2 reduction; no cytokines directly reported	HMGB1 upregulates HPGD via RAGE-MEK; potential therapeutic target.
**Pesold VV et al., 2023 [45]**	HMGB1 significantly upregulated	Proteomic study using clinical samples	Western blot: CRSsNP (n = 25), Controls (n = 23);	N/A	MIP-1β, FOXP3	HMGB1 identified as non-invasive biomarker for CRSsNP endotyping.
**Bellussi LM et al., 2016 [46]**	RAGE activity highlighted; inhibited by glycyrrhetic acid	Review and experimental study;	10 biopsies from CRSsNP patients, 31 from CRSwNP patients, and 3 healthy nasal mucosa samples	HMGB1 increased in severe nasal obstruction	TNF-α, IL-5, IL-8	Glycyrrhetic acid inhibits HMGB1; therapeutic potential for severe cases.
**Min HJ et al., 2015 [53]**	N/A	Cross-sectional study analyzing nasal lavage samples.	38 CRS patients; Total 63 nasal lavage samples	HMGB1 levels correlate with inflammation severity	IL-8 only significant correlation	HMGB1 correlates strongly with inflammation severity in CRS.
**Chen D et al., 2017 [65]**	SIRT6 downregulated; modulates HMGB1	Experimental study (in vitro and ex vivo analysis)	N/A	N/A	N/A	SIRT6 depletion triggers HMGB1 release; glycyrrhetinic acid mitigates by enhancing Sirt6 expression;
**Min HJ & Kim KS, 2021 [60]**	Higher HMGB1 in ECRSwNP compared to NECRSwNP	Comparative experimental study	26 nasal polyp samples from patients with ECRSwNP and NECRSwNP	HMGB1 elevated in severe ECRSwNP	N/A	HMGB1 distinguishes ECRSwNP from NECRSwNP; potential biomarker.
**Lee SH et al., 2021 [55]**	RAGE-mediated signaling; ECM remodeling	Experimental study (in vitro analysis) using human nasal fibroblast cultures	8 patients	HMGB1 promotes tissue remodeling severity	α-SMA, fibronectin, collagen	HMGB1 drives fibroblast differentiation and ECM production via RAGE.
**Xu J et al., 2018 [62]**	TLR9 activation increases BAFF via HMGB1	Experimental study (ex vivo and in vitro analysis)	17 patients with CRSwNP; 10 patients with CRSsNP; 18 control subjects	HMGB1 correlated with BAFF levels in NP tissues	Type-I IFN, BAFF	TLR9 activation via HMGB1 amplifies BAFF and inflammation; HMGB1 plays a role in TLR9-mediated immune activation.
**Dzaman K et al., 2015 [51]**	RAGE overexpressed; linked to inflammation	Comparative observational study	25 patients with recalcitrant CRSwNP; 26 control subjects	RAGE expression correlates with disease severity	N/A	RAGE linked to severe recalcitrant CRSwNP; therapeutic target potential.
**Hao W et al., 2021 [56]**	miR-1287-5p inhibits HMGB1 and EMT	In vitro experimental study using HNEC cultures	N/A	N/A	IL-6, IL-8, TNF-α	miR-1287-5p inhibits HMGB1 and EMT; Inhibition of HMGB1 using Glycyrrhizin suppressed inflammatory cytokines and EMT in nasal epithelial cells.
**Xie Y et al., 2021 [48]**	HMGB1 as DAMP via necroptosis; RAGE/TLR4	Comparative experimental study	48 control subjects; 34 patients with ECRSwNP; 35 patients with NECRSwNP	HMGB1 release correlates with neutrophilic inflammation	IL-1a; IL-6; IL-8, CXCL1, TNF-α, IFN-γ	HMGB1 levels are significantly elevated in CRSwNP tissues; Necroptosis triggers HMGB1 release; promotes neutrophilic inflammation.
**Choi T et al., 2024 [57]**	HMGB1 role in EMT via TGF-β, Wnt	Review study	N/A	HMGB1 drives EMT in severe CRS cases	TNF-α, TGF-β	HMGB1 drives EMT in CRS, promoting tissue remodeling.
**Taziki MH et al., 2019 [54]**	TLR4 correlation with HMGB1	Basic science study using qRT-PCR	26 CRS patients; 26 control subjects	HMGB1-TLR4 correlation with inflammation	N/A	HMGB1-TLR4 synergistically amplifies inflammation in CRSwNP.
**Yang P et al., 2019 [58]**	PPAR-γ inhibition of HMGB1-driven EMT	Experimental study (ex vivo and in vitro analysis)	18 ECRSwNP tissue samples; 12 control nasal mucosa samples	HMGB1-driven EMT correlates with ECRSwNP severity	N-cadherin, vimentin	PPAR-γ agonists inhibit HMGB1-driven EMT in ECRSwNP.
**Dzaman K et al., 2015 [52]**	RAGE overexpression in recalcitrant CRSsNP	Comparative observational study	37 CRSsNP patients; 26 control subjects	RAGE overexpression correlates with severe inflammation	N/A	RAGE expression correlates with disease severity in CRSsNP. HMGB1 may not be a key differentiating factor in CRSsNP pathogenesis.
**Cho HJ & Kim CH, 2018 [66]**	Hypoxia induces HMGB1 via ROS	Review and experimental study	In vitro studies were conducted HNECs	Hypoxia-induced HMGB1 linked to inflammation severity	MUC5AC, VEGF, IL-8	Hypoxia induces HMGB1 release, amplifying CRS inflammation; HMGB1 secretion under hypoxia is ROS-dependent, as it was blocked by the ROS scavenger NAC

**Table 3:** Summary of studies evaluating HMGB1 in allergic rhinitis (AR), with details on experimental approaches, subject characteristics, and relevant outcomes.

Authors	Key Findings	Implications
**Zhong N et al. [67]**	HMGB1 and IL-33 expression were significantly higher in AR nasal mucosa; linked to allergen-induced inflammation.	Suggests HMGB1 and IL-33 as key players in AR inflammation; potential therapeutic targets.
**Zhu X et al. [68]**	HMGB1/TLR4 pathway correlates positively with pro-inflammatory ILs (IL-4, IL-5, IL-13, IL-17A) and negatively with IL-10 in AR.	Highlights HMGB1/TLR4 pathway’s role in AR pathogenesis and its potential for immunotherapy.
**Zhu YM et al. [70]**	miR-141-3p negatively regulates HMGB1 expression, reducing mucus production and apoptosis in LPS-treated cells.	Demonstrates a regulatory axis (miR-141-3p/HMGB1) as a possible therapeutic target for AR.
**Xing X et al. [69]**	Serum HMGB1 and HMGB2 levels correlate with inflammatory markers and clinical severity; potential biomarkers for AR.	Supports HMGB1 and HMGB2 as diagnostic and prognostic biomarkers in AR management.
**Wu S et al. [43]**	HMGB1 contributes to immune dysregulation and inflammation in AR; inhibitors show therapeutic promise in AR models.	Suggests targeting HMGB1 with inhibitors as a novel therapeutic strategy for AR.

**Table 4:** Studies addressing the involvement of HMGB1 in both CRS and AR. The table includes comparative findings and shared mechanisms highlighted by each study.

Pathway	Key Cytokines	Relevance to Disease	Role of HMGB1
**Th1**	IFN-γ, TNF-α	Associated with non-eosinophilic CRS; neutrophilic inflammation	Amplifies cytokine release and neutrophil recruitment through TLR4/RAGE pathways
**Th2**	IL-4, IL-5, IL-13	Key driver in CRSwNP and AR; eosinophilic inflammation, IgE production	Promotes Th2 polarization, enhances eosinophil recruitment, and IgE production
**Th17**	IL-17A, IL-22	Linked to epithelial barrier dysfunction and severe inflammation	Drives epithelial barrier dysfunction and amplifies IL-17A-mediated inflammation

**Table 5:** Summary of non-specific or general studies on HMGB1 in upper airway inflammation, not restricted to CRS or AR but providing relevant mechanistic or translational insights.

Common Findings	Relevant Studies
HMGB1 is consistently upregulated in CRS and AR patients compared to controls.	Pesold VV et al., 2023 [45]; Min HJ et al., 2015 [53]; Bellussi LM et al., 2016 [46];
HMGB1 plays a role in EMT, contributing to tissue remodeling.	Lee SH et al., 2021 [55]; Choi T et al., 2024 [57]; Hao W et al., 2021 [56]; Yang P et al., 2019 [58];
In eosinophilic CRS (ECRSwNP), HMGB1 levels are significantly higher than in non-eosinophilic forms.	Min HJ & Kim KS, 2021 [60]; Xie Y et al., 2021 [48]
Glycyrrhizin and glycyrrhetinic acid reduce HMGB1-related inflammation and show therapeutic potential.	Bellussi LM et al., 2016 [46]; Hao W et al., 2021 [56]; Chen D et al., 2017 [65];
HMGB1-TLR4 and HMGB1-TLR9 signaling pathways are linked to increased cytokine production.	Taziki MH et al., 2019 [54]; Xu J et al., 2018 [62]
Hypoxia enhances HMGB1 release, further exacerbating inflammation.	Cho HJ & Kim CH, 2018 [66]
miRNAs (e.g., miR-1287-5p and miR-141-3p) regulate HMGB1 expression and inflammatory responses.	Hao W et al., 2021 [56]; Zhu YM et al., 2020 [70]
HMGB1 may serve as a potential biomarker for differentiating CRS subtypes.	Min HJ & Kim KS, 2021 [60]; Dzaman K et al., 2015 [52]
HMGB1-driven inflammation correlates with disease severity	Pesold VV et al., 2023 [45]; Xing X et al., 2023 [69]; Yang P et al., 2019 [58]; Dzaman K et al., 2015 [51]; Cho HJ & Kim CH, 2018 [66];

## Data Availability

No new data were created or analyzed in this study.

## Abbreviations

The following abbreviations are used in this manuscript:

AP-1	Activator Protein 1
AR	Allergic Rhinitis
BAFF	B-cell Activating Factor
CRP	C-Reactive Protein
CRS	Chronic Rhinosinusitis
CRSwNP	Chronic Rhinosinusitis with Nasal Polyps
CRSsNP	Chronic Rhinosinusitis without Nasal Polyps
CXCL1	C-X-C Motif Chemokine Ligand 1
ECM	Extracellular Matrix
ECRSwNP	Eosinophilic Chronic Rhinosinusitis with Nasal Polyps
EMT	Epithelial–Mesenchymal Transition
ERK	Extracellular Signal-Regulated Kinase
GA	Glycyrrhetinic Acid
GSDMD	Gasdermin D
HIF-1α	Hypoxia-Inducible Factor 1 Alpha
HMGB1	High Mobility Group Box 1
HPGD	15-Hydroxyprostaglandin Dehydrogenase
HNEC	Human Nasal Epithelial Cells
ICAM-1	Intercellular Adhesion Molecule 1
IFN-γ	Interferon Gamma
IgE	Immunoglobulin E
IL-1α	Interleukin 1 Alpha
IL-1β	Interleukin 1 Beta
IL-4	Interleukin 4
IL-5	Interleukin 5
IL-6	Interleukin 6
IL-8	Interleukin 8
IL-10	Interleukin 10
IL-13	Interleukin 13
IL-17A	Interleukin 17A
JNK	c-Jun N-terminal Kinase
LPS	Lipopolysaccharides
MAPK	Mitogen-Activated Protein Kinase
MCP-1	Monocyte Chemoattractant Protein 1
MEK	Mitogen-Activated Protein Kinase
MLKL	Mixed Lineage Kinase Domain-Like Protein
MUC5AC	Mucin 5AC
NAC	N-Acetyl Cysteine
NF-κB	Nuclear Factor Kappa-Light-Chain-Enhancer of Activated B Cells
NECRSwNP	Non-Eosinophilic Chronic Rhinosinusitis with Nasal Polyps
NETs	Neutrophil Extracellular Traps
NLS	Nuclear Localization Signals
NLRP3	NOD-, LRR-, and Pyrin Domain-Containing Protein 3
PAI-1	Plasminogen Activator Inhibitor-1
PGE2	Prostaglandin E2
PPAR-γ	Peroxisome Proliferator-Activated Receptor Gamma
PRRs	Pattern Recognition Receptors
RAGE	Receptor for Advanced Glycation End Products
RIPK3	Receptor-Interacting Protein Kinase 3
ROG	Rosiglitazone
ROS	Reactive Oxygen Species
RNS	Reactive Nitrogen Species
SIRT6	Sirtuin 6
SNAI1	Snail Family Transcriptional Repressor 1
SNOT-20	Sino-Nasal Outcome Test-20
TGF-β	Transforming Growth Factor Beta
Th1	T-helper 1 Cells
Th2	T-helper 2 Cells
Th17	T-helper 17 Cells
TLR	Toll-Like Receptor
TLR2	Toll-Like Receptor 2
TLR4	Toll-Like Receptor 4
TLR9	Toll-Like Receptor 9
TNF-α	Tumor Necrosis Factor Alpha
VCAM-1	Vascular Cell Adhesion Molecule 1
VEGF	Vascular Endothelial Growth Factor
Wnt	Wnt Signaling Pathway
ZO-1	Zona Occludens-1

## References

[1] Dumitriu I.E., Baruah P., Manfredi A.A., Bianchi M.E., Rovere-Querini P. (2005). HMGB1: An immune odyssey. Discov. Med..

[2] Andersson U., Erlandsson-Harris H., Yang H., Tracey K.J. (2002). HMGB1 as a DNA-binding cytokine. J. Leukoc. Biol..

[3] Lange S.S., Vasquez K.M. (2009). HMGB1: The jack-of-all-trades protein is a master DNA repair mechanic. Mol. Carcinog..

[4] Nightingale K., Dimitrov S., Reeves R., Wolffe A.P. (1996). Evidence for a shared structural role for HMG1 and linker histones B4 and H1 in organizing chromatin. EMBO J..

[5] van Beijnum J.R., Buurman W.A., Griffioen A.W. (2008). Convergence and amplification of toll-like receptor (TLR) and receptor for advanced glycation end products (RAGE) signaling pathways via high mobility group B1 (HMGB1). Angiogenesis.

[6] Yang S., Xu L., Yang T., Wang F. (2014). High-mobility group box-1 and its role in angiogenesis. J. Leukoc. Biol..

[7] Chen R., Kang R., Tang D. (2022). The mechanism of HMGB1 secretion and release. Exp. Mol. Med..

[8] Yang H., Wang H., Andersson U. (2020). Targeting Inflammation Driven by HMGB1. Front. Immunol..

[9] Andersson U., Yang H., Harris H. (2018). High-mobility group box 1 protein (HMGB1) operates as an alarmin outside as well as inside cells. Semin. Immunol..

[10] Yang H., Wang H., Czura C.J., Tracey K.J. (2005). The cytokine activity of HMGB1. J. Leukoc. Biol..

[11] Chen G., Ward M.F., Sama A.E., Wang H. (2004). Extracellular HMGB1 as a proinflammatory cytokine. J. Interf. Cytokine Res..

[12] Erlandsson Harris H., Andersson U. (2004). Mini-review: The nuclear protein HMGB1 as a proinflammatory mediator. Eur. J. Immunol..

[13] Sun N.K., Chao C.C. (2005). The cytokine activity of HMGB1—Extracellular escape of the nuclear protein. Chang Gung Med. J..

[14] Bianchi M.E., Manfredi A.A. (2007). High-mobility group box 1 (HMGB1) protein at the crossroads between innate and adaptive immunity. Immunol. Rev..

[15] Ulloa L., Messmer D. (2006). High-mobility group box 1 (HMGB1) protein: Friend and foe. Cytokine Growth Factor Rev..

[16] Kang R., Chen R., Zhang Q., Hou W., Wu S., Cao L., Huang J., Yu Y., Fan X.-G., Yan Z. (2014). HMGB1 in health and disease. Mol. Asp. Med..

[17] Chen D., Bellussi L.M., Passali D., Chen L. (2013). LPS may enhance expression and release of HMGB1 in human nasal epithelial cells in vitro. Acta Otorhinolaryngol. Ital..

[18] Chen D., Mao M., Bellussi L.M., Passali D., Chen L. (2014). Increase of high mobility group box chromosomal protein 1 in eosinophilic chronic rhinosinusitis with nasal polyps. Int. Forum Allergy Amp. Rhinol..

[19] Ferrara M., Chialli G., Ferreira L.M., Ruggieri E., Careccia G., Preti A., Piccirillo R., Bianchi M.E., Sitia G., Venereau E. (2020). Oxidation of HMGB1 Is a Dynamically Regulated Process in Physiological and Pathological Conditions. Front. Immunol..

[20] Bianchi M.E., Crippa M.P., Manfredi A.A., Mezzapelle R., Rovere Querini P., Venereau E. (2017). High-mobility group box 1 protein orchestrates responses to tissue damage via inflammation, innate and adaptive immunity, and tissue repair. Immunol. Rev..

[21] Singh H., Agrawal D.K. (2022). Therapeutic Potential of Targeting the HMGB1/RAGE Axis in Inflammatory Diseases. Molecules.

[22] Paudel Y.N., Angelopoulou E., Piperi C., Balasubramaniam V.R., Othman I., Shaikh M.F. (2019). Enlightening the role of high mobility group box 1 (HMGB1) in inflammation: Updates on receptor signalling. Eur. J. Pharmacol..

[23] Rauvala H., Rouhiainen A. (2007). RAGE as a receptor of HMGB1 (Amphoterin): Roles in health and disease. Curr. Mol. Med..

[24] Rouhiainen A., Kuja-Panula J., Tumova S., Rauvala H. (2013). RAGE-mediated cell signaling. Methods Mol. Biol..

[25] Ibrahim Z.A., Armour C.L., Phipps S., Sukkar M.B. (2013). RAGE and TLRs: Relatives, friends or neighbours?. Mol. Immunol..

[26] Fiuza C., Bustin M., Talwar S., Tropea M., Gerstenberger E., Shelhamer J.H., Suffredini A.F. (2003). Inflammation-promoting activity of HMGB1 on human microvascular endothelial cells. Blood.

[27] Treutiger C.J., Mullins G.E., Johansson A.M., Rouhiainen A., Rauvala H.M.E., Erlandsson-Harris H., Andersson U., Yang H., Tracey K.J., Andersson J. (2003). High mobility group 1 B-box mediates activation of human endothelium. J. Intern. Med..

[28] Bernard N.J. (2018). HMGB1+ platelet microparticles damage the endothelium. Nat. Rev. Rheumatol..

[29] Leng Y., Chen R., Chen R., He S., Shi X., Zhou X., Zhang Z., Chen A.F. (2020). HMGB1 mediates homocysteine-induced endothelial cells pyroptosis via cathepsin V-dependent pathway. Biochem. Biophys. Res. Commun..

[30] Yang H., Wang H., Chavan S.S., Andersson U. (2015). High Mobility Group Box Protein 1 (HMGB1): The Prototypical Endogenous Danger Molecule. Mol. Med..

[31] Lee S.A., Kwak M.S., Kim S., Shin J.S. (2014). The role of high mobility group box 1 in innate immunity. Yonsei Med. J..

[32] Yanai H., Ban T., Taniguchi T. (2012). High-mobility group box family of proteins: Ligand and sensor for innate immunity. Trends Immunol..

[33] Yang H., Tracey K.J. (2010). Targeting HMGB1 in inflammation. Biochim. Biophys. Acta (BBA)-Gene Regul. Mech..

[34] Liu J., Song K., Lin B., Chen Z., Zuo Z., Fang Y., He Q., Yao X., Liu Z., Huang Q. (2024). HMGB1 promotes neutrophil PD-L1 expression through TLR2 and mediates T cell apoptosis leading to immunosuppression in sepsis. Int. Immunopharmacol..

[35] Lou H., Zhang N., Bachert C., Zhang L. (2018). Highlights of eosinophilic chronic rhinosinusitis with nasal polyps in definition, prognosis, and advancement. Int. Forum Allergy Rhinol..

[36] Cao P.P., Wang Z.C., Schleimer R.P., Liu Z. (2019). Pathophysiologic mechanisms of chronic rhinosinusitis and their roles in emerging disease endotypes. Ann. Allergy Asthma Immunol..

[37] Vanderhaegen T., Gengler I., Dendooven A., Chenivesse C., Lefèvre G., Mortuaire G. (2022). Eosinophils in the Field of Nasal Polyposis: Towards a Better Understanding of Biologic Therapies. Clin. Rev. Allergy Immunol..

[38] Tsuda T., Suzuki M., Kato Y., Kidoguchi M., Kumai T., Fujieda S., Sakashita M. (2024). The current findings in eosinophilic chronic rhinosinusitis. Auris Nasus Larynx.

[39] Bachert C., Akdis C.A. (2016). Phenotypes and Emerging Endotypes of Chronic Rhinosinusitis. J. Allergy Clin. Immunol. Pract..

[40] Wang H., Pan L., Liu Z. (2019). Neutrophils as a Protagonist and Target in Chronic Rhinosinusitis. Clin. Exp. Otorhinolaryngol..

[41] Siddiqui Z.A., Walker A., Pirwani M.M., Tahiri M., Syed I. (2022). Allergic rhinitis: Diagnosis and management. Br. J. Hosp. Med..

[42] Ponda P., Carr T., Rank M.A., Bousquet J. (2023). Nonallergic Rhinitis, Allergic Rhinitis, and Immunotherapy: Advances in the Last Decade. J. Allergy Clin. Immunol. Pract..

[43] Wu S., Yu Y., Zheng Z., Cheng Q. (2023). High mobility group box-1: A potential therapeutic target for allergic rhinitis. Eur. J. Med. Res..

[44] Bellussi L.M., Iosif C., Sarafoleanu C., Jianu E., Duda R., Panaitescu E., Passali F.M., Passali D. (2013). Are HMGB1 protein expression and secretion markers of upper airways inflammatory diseases?. J. Biol. Regul. Homeost. Agents.

[45] Pesold V.V., Wendler O., Morgenthaler L., Gröhn F., Mueller S.K. (2023). Analysis of CRSsNP Proteome Using a Highly Multiplexed Approach in Nasal Mucus. Am. J. Rhinol. Allergy.

[46] Bellussi L.M., Cocca S., Chen L., Passali F.M., Sarafoleanu C., Passali D. (2016). Rhinosinusal Inflammation and High Mobility Group Box 1 Protein: A New Target for Therapy. ORL J. Otorhinolaryngol. Relat. Spec..

[47] Dyer K.D., Rosenberg H.F. (2015). Physiologic concentrations of HMGB1 have no impact on cytokine-mediated eosinophil survival or chemotaxis in response to Eotaxin-2 (CCL24). PLoS ONE.

[48] Xie Y., Li M., Chen K., Zhu H., Tang M., Zhou C., Zheng Y., Wen J., Han M., Zhang J. (2021). Necroptosis Underlies Neutrophilic Inflammation Associated with the Chronic Rhinosinusitis with Nasal Polyps (CRSwNP). J. Inflamm. Res..

[49] Ciprandi G., Bellussi L.M., Passali G.C., Damiani V., Passali D. (2020). HMGB1 in nasal inflammatory diseases: A reappraisal 30 years after its discovery. Expert Rev. Clin. Immunol..

[50] Bellussi L.M., Cocca S., Passali G.C., Passali D. (2017). HMGB1 in the Pathogenesis of Nasal Inflammatory Diseases and its Inhibition as New Therapeutic Approach: A Review from the Literature. Int. Arch. Otorhinolaryngol..

[51] Dzaman K., Szczepanski M.J., Molinska-Glura M., Krzeski A., Zagor M. (2015). Expression of the receptor for advanced glycation end products, a target for high mobility group box 1 protein, and its role in chronic recalcitrant rhinosinusitis with nasal polyps. Arch. Immunol. Ther. Exp..

[52] Dzaman K., Zagor M., Molinska-Glura M., Krzeski A. (2015). High motility group box 1 (HMGB1) protein and its receptor for advanced glycation end products (RAGE) expression in chronic rhinosinusitis without nasal polyps. Folia Histochem. Cytobiol..

[53] Min H.J., Kim S.J., Kim T.H., Chung H.J., Yoon J.H., Kim C.H. (2015). Level of secreted HMGB1 correlates with severity of inflammation in chronic rhinosinusitis. Laryngoscope.

[54] Taziki M.H., Azarhoush R., Taziki M.M., Naghavi-Alhosseini M., Javid N., Davoodi H. (2019). Correlation Between HMGB1 and TLR4 Expression in Sinonasal Mucosa in Patients With Chronic Rhinosinusitis. Ear Nose Throat J..

[55] Lee S.H., Cho J.H., Park J.H., Cho J.S., Lee H.M. (2021). High Mobility Group Box Chromosomal Protein-1 Induces Myofibroblast Differentiation and Extracellular Matrix Production via RAGE, p38, JNK and AP-1 Signaling Pathways in Nasal Fibroblasts. Am. J. Rhinol. Allergy.

[56] Hao W., Zhu Y., Guo Y., Wang H. (2021). miR-1287-5p upregulation inhibits the EMT and pro-inflammatory cytokines in LPS-induced human nasal epithelial cells (HNECs). Transpl. Immunol..

[57] Choi T., Ryu S., Bae J.S., Yoo S.H., Mo J.H. (2024). Epithelial-Mesenchymal Transition in Chronic Rhinosinusitis. J. Rhinol..

[58] Yang P., Chen S., Zhong G., Kong W., Wang Y. (2019). Agonist of PPAR-γ Reduced Epithelial-Mesenchymal Transition in Eosinophilic Chronic Rhinosinusitis with Nasal Polyps via Inhibition of High Mobility Group Box1. Int. J. Med. Sci..

[59] Shimizu S., Kouzaki H., Kato T., Tojima I., Shimizu T. (2016). HMGB1-TLR4 signaling contributes to the secretion of interleukin 6 and interleukin 8 by nasal epithelial cells. Am. J. Rhinol. Allergy.

[60] Min H.J., Kim K.S. (2020). Expression Pattern of HMGB1 Differs Between Eosinophilic Chronic Rhinosinusitis with Nasal Polyp and Non-Eosinophilic Chronic Rhinosinusitis With Nasal Polyp: A Preliminary Study. Am. J. Rhinol. Allergy.

[61] Chen S., Chen J., Chen J., Wang Y. (2023). Altered expression of 15-hydroxyprostaglandin dehydrogenase in chronic rhinosinusitis with nasal polyps. Lin Chuang Er Bi Yan Hou Tou Jing Wai Ke Za Zhi (J. Clin. Otorhinolaryngol. Head Neck Surg.).

[62] Xu J., Lee J.-W., Park S.-K., Lee S.-B., Yoon Y.-H., Yeon S.-H., Rha K.-S., Choi J.-A., Song C.-H., Kim Y.M. (2018). Toll-like receptor 9 ligands increase type I interferon induced B-cell activating factor expression in chronic rhinosinusitis with nasal polyposis. Clin. Immunol..

[63] Cavone L., Cuppari C., Manti S., Grasso L., Arrigo T., Calamai L., Salpietro C., Chiarugi A. (2015). Increase in the Level of Proinflammatory Cytokine HMGB1 in Nasal Fluids of Patients with Rhinitis and its Sequestration by Glycyrrhizin Induces Eosinophil Cell Death. Clin. Exp. Otorhinolaryngol..

[64] Passali D., Cappello C., Passali G.C., Cingi C., Sarafoleanu C., Bellussi L.M. (2017). Nasal Muco-ciliary transport time alteration: Efficacy of 18 B Glycyrrhetinic acid. Multidiscip. Respir. Med..

[65] Chen D., Bellussi L.M., Cocca S., Wang J., Passali G.C., Hao X., Chen L., Passali D. (2017). Glycyrrhetinic acid suppressed hmgb1 release by up-regulation of Sirt6 in nasal inflammation. J. Biol. Regul. Homeost. Agents.

[66] Cho H.J., Kim C.H. (2018). Oxygen matters: Hypoxia as a pathogenic mechanism in rhinosinusitis. BMB Rep..

[67] Zhong N., Luo Q., Huang X., Yu J., Ye J., Zhang J. (2022). High Mobility Group Box-1 Protein and Interleukin 33 Expression in Allergic Rhinitis. ORL J. Otorhinolaryngol. Relat. Spec..

[68] Zhu X., Cong J., Yang B., Sun Y. (2020). Association analysis of high-mobility group box-1 protein 1 (HMGB1)/toll-like receptor (TLR) 4 with nasal interleukins in allergic rhinitis patients. Cytokine.

[69] Xing X., Wang H. (2023). Correlation of serum HMGB1 and HMGB2 levels with clinical symptoms in allergic rhinitis children. Medicine.

[70] Zhu Y.M., Wu F., Zhou J.Y. (2020). Analysis the effect of miR-141-3p/HMGB1 in LPS-induced mucus production and the apoptosis in nasal epithelial cells. Kaohsiung J. Med. Sci..

[71] Ohwada K., Konno T., Kohno T., Nakano M., Ohkuni T., Miyata R., Kakuki T., Kondoh M., Takano K., Kojima T. (2021). Effects of HMGB1 on Tricellular Tight Junctions via TGF-β Signaling in Human Nasal Epithelial Cells. Int. J. Mol. Sci..

[72] Zheng J., Wei X., Zhan J.B., Jiang H.Y. (2017). High mobility group box1 contributes to hypoxia-induced barrier dysfunction of nasal epithelial cells. Lin Chuang Er Bi Yan Hou Tou Jing Wai Ke Za Zhi (J. Clin. Otorhinolaryngol. Head Neck Surg.).

[73] Min H.J., Kim J.H., Yoo J.E., Oh J.H., Kim K.S., Yoon J.H., Kim C.H. (2017). ROS-dependent HMGB1 secretion upregulates IL-8 in upper airway epithelial cells under hypoxic condition. Mucosal Immunol..

[74] Bianchi M.E. (2009). HMGB1 loves company. J. Leukoc. Biol..

[75] Hreggvidsdottir H.S., Östberg T., Wähämaa H., Schierbeck H., Aveberger A.-C., Klevenvall L., Palmblad K., Ottosson L., Andersson U., Harris H.E. (2009). The alarmin HMGB1 acts in synergy with endogenous and exogenous danger signals to promote inflammation. J. Leukoc. Biol..

